# Complex Ebstein's Anomaly in an 86-Year-Old Iranian Man: A Case Report

**Published:** 2017-01

**Authors:** Bahieh Moradi, Farideh Roshanali

**Affiliations:** *Day General Hospital, Tehran, Iran.*

**Keywords:** *Ebstein anomaly*, *Tricuspid valve regurgitation*, *Life expectancy*

## Abstract

Ebstein's anomaly is defined as the significant apical displacement of the tricuspid valve causing tricuspid regurgitation. Although a variety of concomitant lesions have been previously described, we herein introduce an unusual presentation. Our patient was an 86-year-old man with a primary presentation of typical chest pain in the setting of recently diagnosed coronary artery disease with concomitant Ebstein’s anomaly. We found mild-to-moderate tricuspid regurgitation, bicuspid aortic valve, persistent left superior vena cava, and patent foramen ovale. The patient had suffered from chest discomfort on exertion for 2 months with good functional capacity prior to diagnosis. Coronary angiography revealed two-vessel disease. The patient refused surgery. He was treated with medical anti-ischemic therapy. He had good exercise tolerance with relief of chest pain at the latest follow-up.

The features demonstrated in this case report suggest that there may be several adult survivors of complex congenital heart diseases requiring individualized surgical treatment plans.

## Introduction

Ebstein's anomaly is a rare congenital cardiac disease. In the natural history of this congenital disease, only 5% of patients survive beyond the fifth decade.^1^ This anomaly is defined as the significant apical displacement of the tricuspid valve, causing tricuspid regurgitation, reduction of the functional right ventricle, dilatation of the right atrium and right ventricle, and atrial and ventricular arrhythmias.^2^ The most frequent associated defect in adult patients is the atrial septal defect, occurring in 38%, or patent foramen ovale. Persistent left superior vena cava is seen in 9%. Other associated lesions include right aortic arch, aortic coarctation, non-compaction of the left ventricular myocardium, tetralogy of Fallot, and aortopulmonary window.^3^^, ^^4^

## Case Report

An 86-year-old man was referred for echocardiography with a 2-month history of anginal chest pain of New York Heart Association (NYHA) class II without hospital admissions. The patient had a history of cigarette smoking for 20 years and systemic hypertension for 10 years. In addition, he had atrial fibrillation rhythm. 

On examination, the patient showed no signs of cyanosis or clubbing, with O_2_ saturation of about 90% in air room. Chest radiography showed a globe-shaped heart, with normal vascularity and a cardiothoracic ratio of 0.5. Echocardiography revealed features of Ebstein’s anomaly with mild-to-moderate tricuspid regurgitation (Figure 1a) and right ventricular and atrial dilation. The function of the right ventricle was preserved. The ratio between the area of the functional right ventricle and the combined area of both the right atrium and the atrialized right ventricle was 42%. The anterior leaflet of the tricuspid valve was enlarged but pliable with mild tethering, and the septal and posterior leaflets were significantly displaced (about 14 mm/m^2^ for the septal and 16 mm/m^2^ for the posterior leaflet; Figure 1b). The left ventricle was of normal size and thickness, and the ejection fraction was estimated at 45-50% due to wall motion abnormalities in the septal, lateral, and anterior segments. There was a small patent foramen ovale, seen in contrast study (Figure 2). Other interesting echocardiographic findings were bicuspid aortic valve (Figure 3) with no stenosis and only mild regurgitation and dilated coronary sinus because of persistent left superior vena cava. The size of the ascending aorta and aortic arch was normal with no coarctation. Coronary angiography revealed a long left main with 50% distal stenosis and significant proximal lesions in the proximal left anterior descending coronary artery and obtuse marginal.

**Figure 1a:**
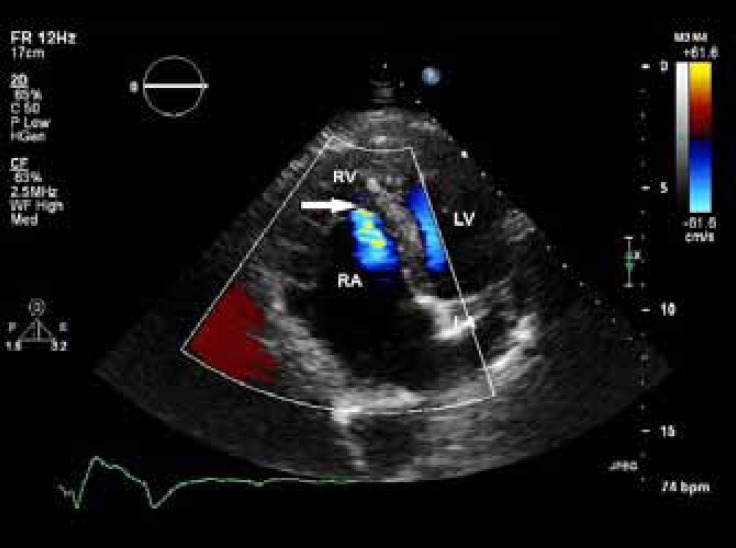
Apical four-chamber view in transthoracic echocardiography, showing Ebstein’s anomaly with mild-to-moderate tricuspid regurgitation (arrow) and right ventricular and atrial dilation

RV, Right ventricle; RA, Right atrium; LV, Left ventricle; LA, Left atrium

**Figure 1b:**
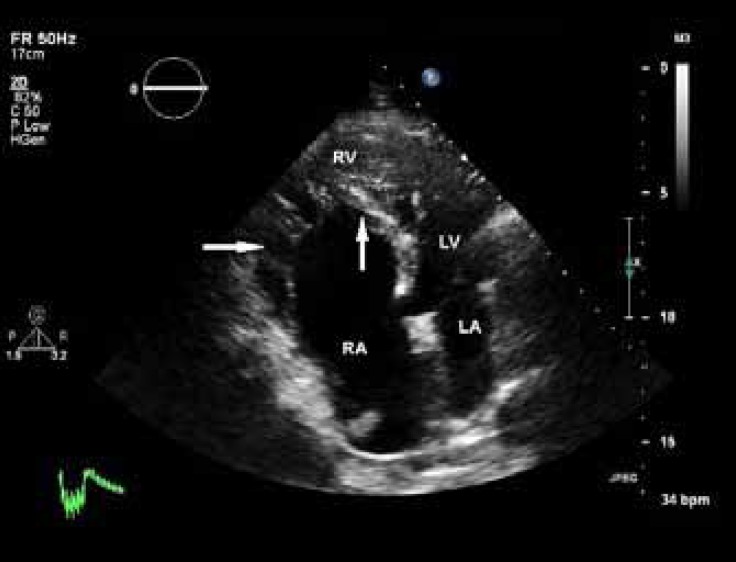
Apical four-chamber view in transthoracic echocardiography, showing an enlarged and pliable anterior tricuspid leaflet with mild tethering (horizontal arrow) and septal tricuspid leaflet with significant apical displacement (vertical arrow)

RV, Right ventricle; RA, Right atrium; LV, Left ventricle; LA, Left atrium

**Figure 2:**
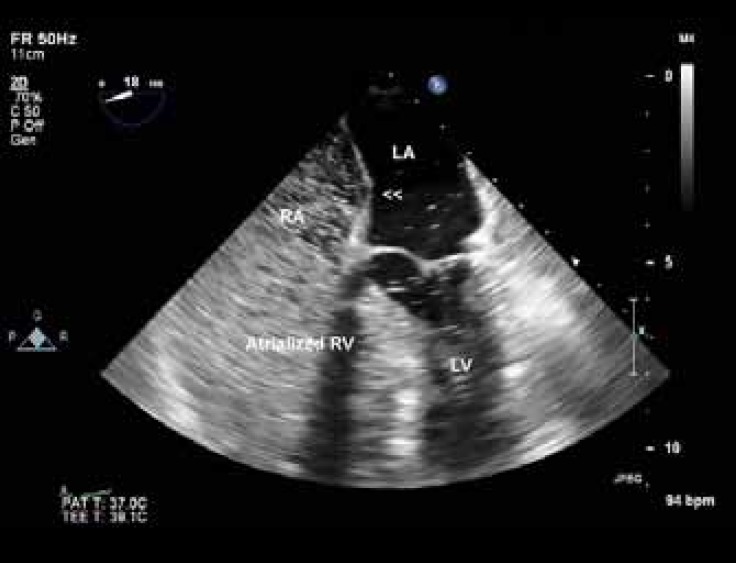
Four-chamber view in transesophageal echocardiography, showing the interatrial septum (arrow heads) with a small patent foramen ovale illustrated in contrast study

RV, Right ventricle; RA, Right atrium; LV, Left ventricle; LA, Left atrium

**Figure 3:**
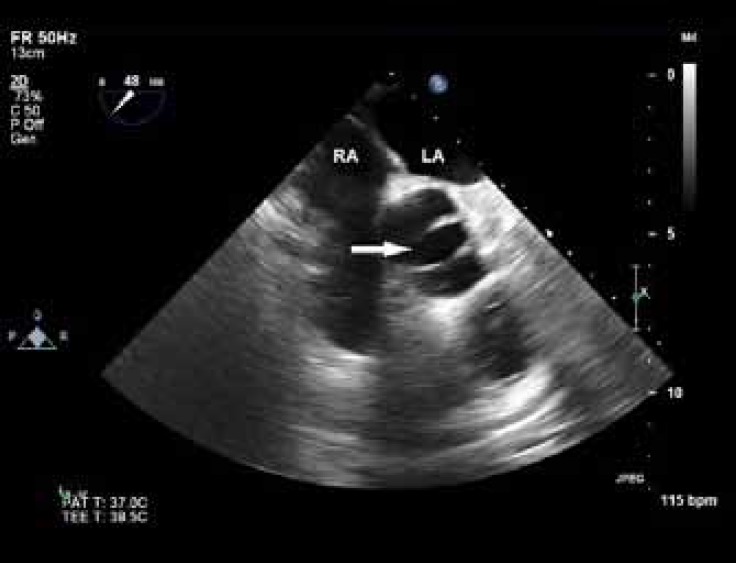
Aortic short-axis view in transesophageal echocardiography, showing the bicuspid aortic valve (arrow): a rare and interesting finding in Ebstein’s anomaly

RA, Right atrium; LA, Left atrium

The patient was candidated for coronary artery bypass grafting surgery, but he refused cardiac surgery. He was, therefore, discharged on medical anti-ischemic therapy (Aspirin, beta blocker, Statin, angiotensin-converting enzyme inhibitor) and anticoagulation therapy with a target international normalized ratio (INR) of 2-2.5 and recommended to have regular follow-ups. 

## Discussion

Ebstein’s anomalies can range from minimal to severe. If the deformity of the tricuspid valve is severe, it may result in profound congestive heart failure in the neonatal period or even in death. At the other end of the spectrum, patients with a mild degree of tricuspid displacement and dysfunction, as was the case in our patient, may remain asymptomatic until late adult life.^4^

A large spectrum of concomitant lesions has been described. Our patient had complex abnormalities as well as a bicuspid aortic valve, which has not been reported to date. Also interestingly in this patient, the coexistence of other illnesses such as systemic hypertension and coronary artery diseases compelled him to refer to a physician.

It should be emphasized the therapeutic approach in these patients may be in question sometimes because the operative indication of asymptomatic Ebstein's anomaly in adult patients has not been clearly defined.^5^ Operative repair of Ebstein's anomaly is performed usually during younger age, although medical management may be used to manage some of the symptoms of heart failure and arrhythmias. Eventually, most patients will require surgery. Observation alone may be advised for asymptomatic and acyanotic patients, mild cardiomegaly, and normal exercise tolerance. Most patients in NYHA classes I and II can be managed medically. Surgery is offered when the patient’s symptoms progress to NYHA class III or IV, increasing cyanosis becomes evident, or if paradoxical embolism occurs.^6^ Many surgeons believe that severe tricuspid regurgitation with moderate right ventricular dysfunction can be the operative indication in adult patients with asymptomatic Ebstein's anomaly, especially when tricuspid valve repair is possible.^5^ Surgery is also advised if there is evidence of decreasing exercise performance by exercise testing, progressive increase in the heart size on chest radiography, progressive right ventricular dilation or reduction of systolic function by echocardiography, or appearance of atrial or ventricular arrhythmias. In borderline situations, the echocardiographic determination of the high probability of tricuspid valve repair makes the decision to proceed earlier with surgery easier.^6^

## Conclusion

There may be several adult survivors of congenital heart diseases which may primarily present with other systemic illnesses. Our patient refused to undergo surgery, but the surgical plan on which we had decided comprised coronary artery bypass grafting with the closure of atrial septal communication. Furthermore, because the patient was acyanotic with good functional capacity, acceptable right ventricular function, and mild-to-moderate tricuspid regurgitation, we did not plan the surgical correction of the associated anomalies.

## Notes:

***This paper should be cited as:*** Moradi B, Roshanali F. Complex Ebstein's Anomaly in an 86-Year-Old Iranian Man: A Case Report. J Teh Univ Heart Ctr 2017;12(1):39-41.
